# Supplementary figures and images for: Pulmonary involvement in human visceral leishmaniasis: Clinical and tomographic evaluation
Source: PLoS One. 2020 Jan 30;15(1):e0228176. doi: 10.1371/journal.pone.0228176 (PMC6992183; doi:10.1371/journal.pone.0228176)

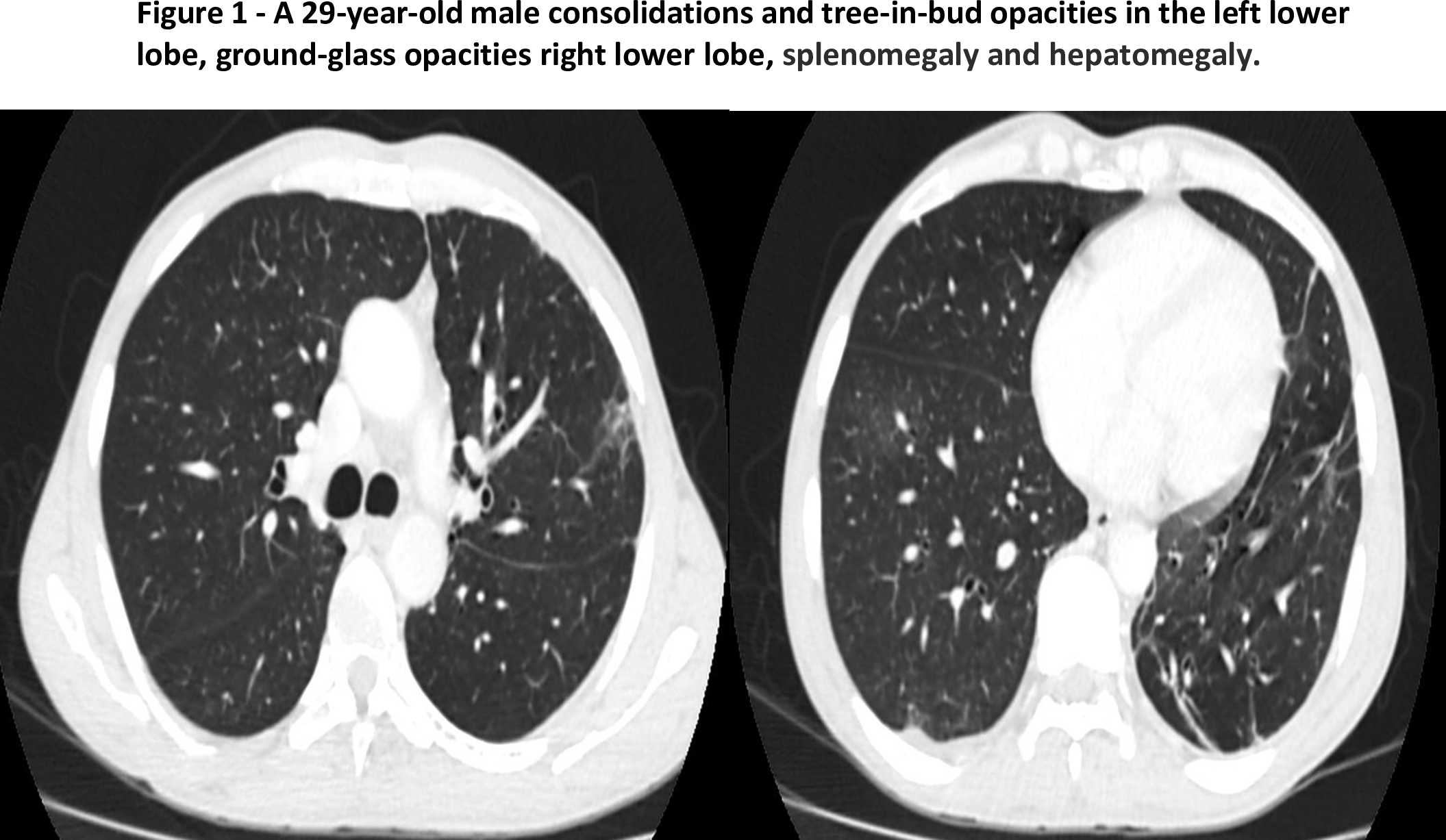

Supplement: S1 Fig — (TIF) [file pone.0228176.s001.tif]

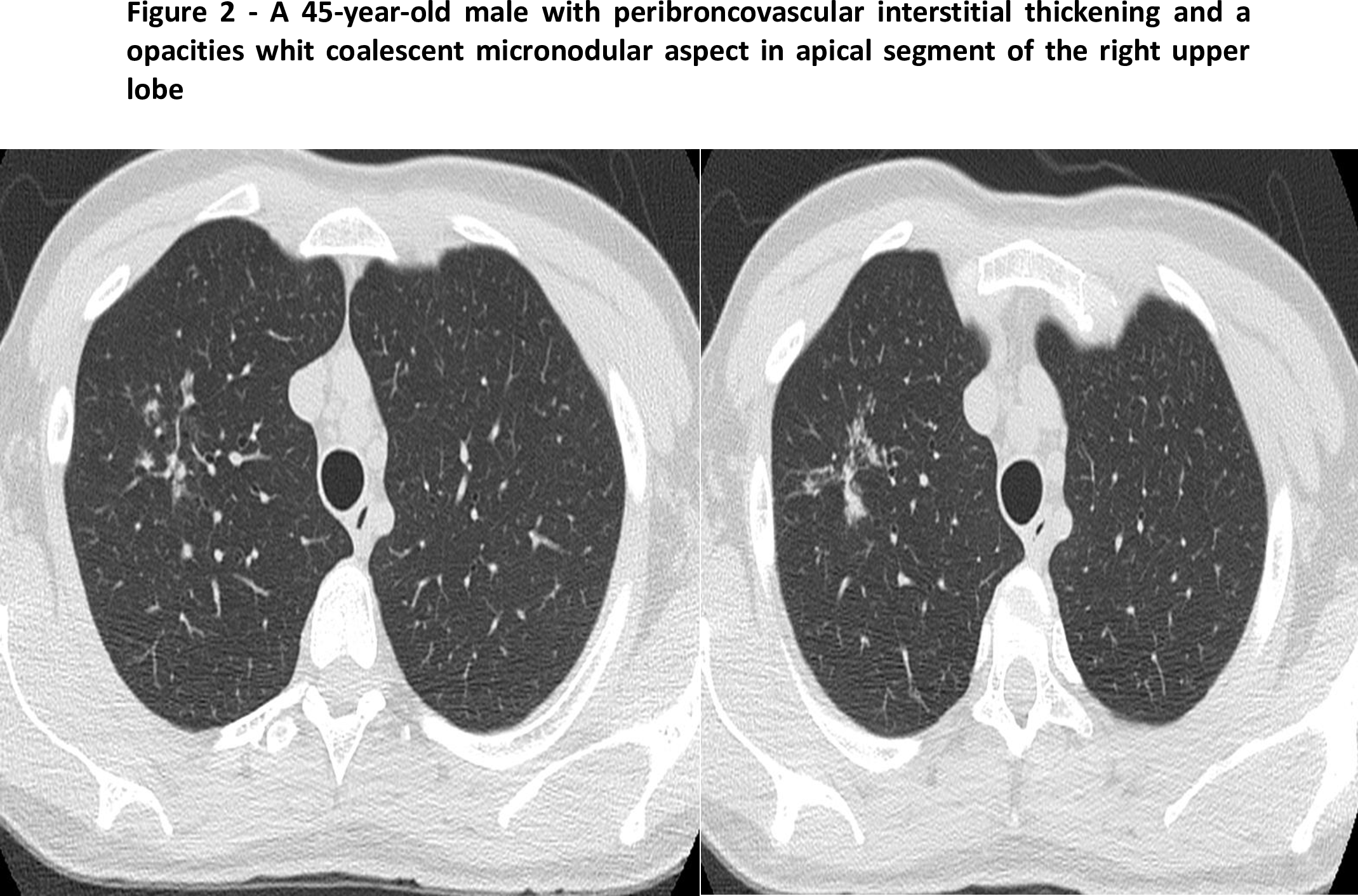

Supplement: S2 Fig — (TIF) [file pone.0228176.s002.tif]

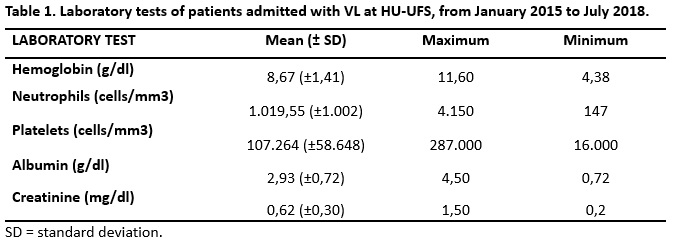

Supplement: S1 Table — (TIF) [file pone.0228176.s003.tif]

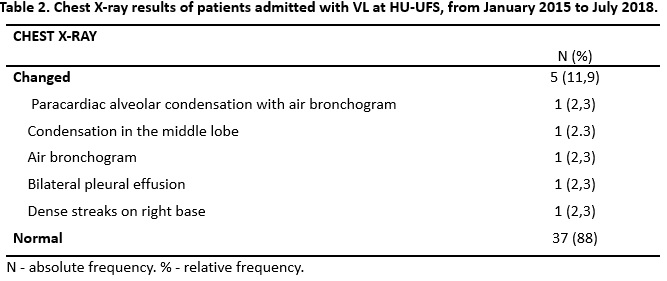

Supplement: S2 Table — (TIF) [file pone.0228176.s004.tif]

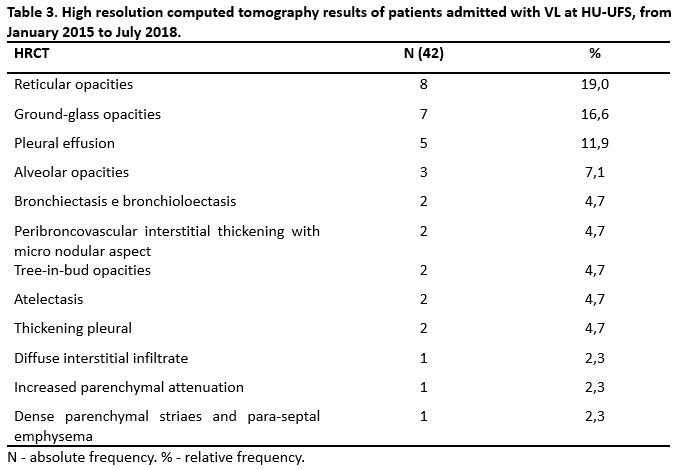

Supplement: S3 Table — (TIF) [file pone.0228176.s005.tif]

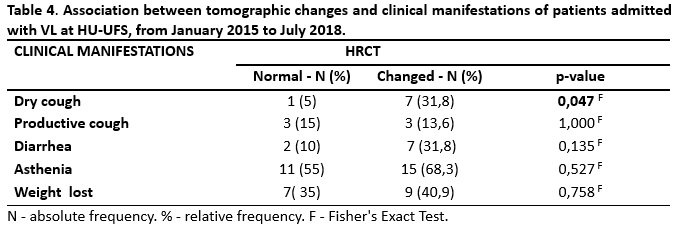

Supplement: S4 Table — (TIF) [file pone.0228176.s006.tif]

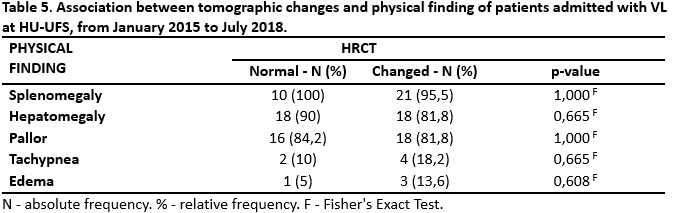

Supplement: S5 Table — (TIF) [file pone.0228176.s007.tif]

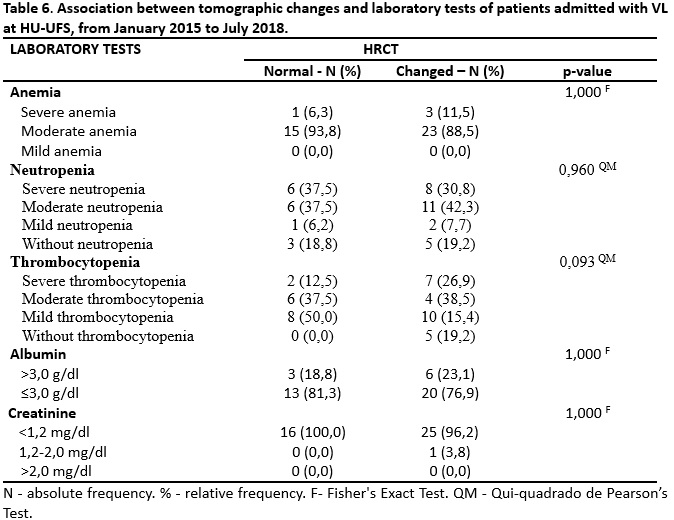

Supplement: S6 Table — (TIF) [file pone.0228176.s008.tif]
