# Supplementary material for: Pulmonary involvement in human visceral leishmaniasis: Clinical and tomographic evaluation
Source: PLoS One. 2020 Jan 30;15(1):e0228176. doi: 10.1371/journal.pone.0228176 (PMC6992183; doi:10.1371/journal.pone.0228176)
Supplement: Attachment — Submitted filename: Comment_PONE-D-19-23416_sept_2019.docx [file pone.0228176.s009.docx]

**Pulmonary Involvement in Human Visceral Leishmaniasis: Clinical and Tomographic**

**Evaluation**

**Manuscript Number: PONE-D-19-23416**

**Comment to the Editor**

Some additional information / modification of the manuscript required as depicted in comments to authors.

**Comment to Authors**

1. As many co-morbidity / past illness may cause pulmonary changes, how changes of lung in radiology was attributed to Visceral Leishmaniasis? As authors mention in conclusion that “cross-sectional design did not allow establishing causal inferences between the studied variables” – this should be conversed in the discussion.
2. What is the exact number of cases of the present study? According to table 5, total cases classified according to severity of anemia is 42, though the total number of cases classified according to severity plus absence of Neutropenia is 39.
3. Inclusion and exclusion criteria (beside “Patients transferred from other health units who had already started treatment at the unit of Origin”) of the present study must be defined.
4. Clinical features / physical finding should be depicted in a separate table.
5. Rationality of correlating some parameters (for example Thrombocytopenia) with HRCT changes of lung should be explained.
6. As bone marrow examination was not done in every case, rationality of the diagnosis by serological investigations should be discussed.
7. Results of radiological finding of the present study should be compared with other similar studies. Was any additional significant findings obtained in the present study?
8. Some characteristic image of changes in the lung as depicted in CT / X ray should be incorporated.
9. Units of biochemical tests in Table 5 should be mentioned.
10. Appropriate symbol for fractional number should be placed.
11. All reference must be quoted according to instructions to authors of the journal.
