# Supplementary material for: Pulmonary involvement in human visceral leishmaniasis: Clinical and tomographic evaluation
Source: PLoS One. 2020 Jan 30;15(1):e0228176. doi: 10.1371/journal.pone.0228176 (PMC6992183; doi:10.1371/journal.pone.0228176)
Supplement: Attachment — Submitted filename: Response to Reviewers.odt [file pone.0228176.s010.odt]

Response to Reviewers

Pulmonary Involvement in Human Visceral Leishmaniasis: Clinical and Tomographic Evaluation
Manuscript Number: PONE-D-19-23416

Some additional information / modification of the manuscript required as depicted in comments to authors.  

Comment to Authors
1.	As many co-morbidity / past illness may cause pulmonary changes, how changes of lung in radiology was attributed to Visceral Leishmaniasis? As authors mention in conclusion that “cross-sectional design did not allow establishing causal inferences between the studied variables” – this should be conversed in the discussion.

RESPONSE:
We evaluated the presence of previous diseases, co-infections and comorbidities. We found HIV / Leishmania coinfection in two patients and in another schistosomiasis mansoni. No other infections were reported by the patient or diagnosed during hospitalization. Four patients had comorbidities: diabetes mellitus (one case), diabetes mellitus associated with systemic arterial hypertension (one case), hypothyroidism (one case), hypothyroidism associated with systemic arterial hypertension (one case). Tomographic changes were present, regardless of past or associated VL diseases, including in children, adolescents and young adults, which corresponds to about 75% of the sample. Considering the importance of this information, we added in the results and made the necessary considerations in the discussion of our manuscript. We added the consideration about the study design and the establishment of causal inferences in the discussion.

2.	What is the exact number of cases of the present study? According to table 5, total cases classified according to severity of anemia is 42, though the total number of cases classified according to severity plus absence of Neutropenia is 39.
RESPONSE: the exact number of cases of the present study is 42. The table was retified. Excuse us for the mistake.
3.	Inclusion and exclusion criteria (beside “Patients transferred from other health units who had already started treatment at the unit of Origin”) of the present study must be defined.
RESPONSE: The hospital where the study was conducted is a referral hospital for Leishmaniasis treatment and receives patients from other hospitals. Patients diagnosed with VL who were already on specific treatment when admitted to HU-UFS did not participate in the study. This clarification had been added in the manuscript.

4.	Clinical features / physical finding should be depicted in a separate table.
RESPONSE: We performed the separation in different tables.

5.	Rationality of correlating some parameters (for example Thrombocytopenia) with HRCT changes of lung should be explained.
RESPONSE: We made that explanation in the discussion

6.	As bone marrow examination was not done in every case, rationality of the diagnosis by serological investigations should be discussed.  
RESPONSE: We made that explanation in the discussion

7.	Results of radiological finding of the present study should be compared with other similar studies. Was any additional significant findings obtained in the present study?
RESPONSE: There is a similar study in the medical literature conducted in past decades (reference 16), which was cited in the text. We made a more detailed comparison of our study with this one previous study.

8.	Some characteristic image of changes in the lung as depicted in CT / X ray should be incorporated.
RESPONSE: We have incorporated images into the manuscript.

9.	Units of biochemical tests in Table 5 should be mentioned.
RESPONSE:  We added Units of biochemical tests in Table


10. Appropriate symbol for fractional number should be placed.
RESPONSE: We have incorporated Appropriate symbol for fractional number.

11.	All reference must be quoted according to instructions to authors of the journal.
RESPONSE: We quoted all reference according to instructions to authors of the journal.
